# Space- and time-resolved investigation on diffusion kinetics of human skin following macromolecule delivery by microneedle arrays

**DOI:** 10.1038/s41598-018-36009-8

**Published:** 2018-12-10

**Authors:** Jonathan C. J. Wei, Isha N. Haridass, Michael L. Crichton, Yousuf H. Mohammed, Stefano C. Meliga, Washington Y. Sanchez, Jeffrey E. Grice, Heather A. E. Benson, Michael S. Roberts, Mark A. F. Kendall

**Affiliations:** 1 Diamantina Institute, Faculty of Medicine, The University of Queensland, Woolloongabba, QLD 4102 Australia; 2 Australian Institute for Bioengineering and Nanotechnology, The University of Queensland, St Lucia, QLD 4072 Australia; 3 School of Pharmacy and Biomedical Sciences, Curtin Health Innovation Research Institute, Curtin University, Bentley, WA 6102 Australia; 4 Institute of Mechanical, Process and Energy Engineering, School of Engineering and Physical Sciences, Heriot-Watt University, Edinburgh, EH14 4AS United Kingdom; 5 Basil Hetzel Institute for Translational Health Research, School of Pharmacy and Medical Sciences, University of South Australia, Adelaide, SA 5011 Australia; 6 Australian National University, Canberra, ACT 0200 Australia

## Abstract

Microscale medical devices are being developed for targeted skin delivery of vaccines and the extraction of biomarkers, with the potential to revolutionise healthcare in both developing and developed countries. The effective clinical development of these devices is dependent on understanding the macro-molecular diffusion properties of skin. We hypothesised that diffusion varied according to specific skin layers. Using three different molecular weights of rhodamine dextran (RD) (MW of 70, 500 and 2000 kDa) relevant to the vaccine and therapeutic scales, we deposited molecules to a range of depths (0–300 µm) in *ex vivo* human skin using the Nanopatch device. We observed significant dissipation of RD as diffusion with 70 and 500 kDa within the 30 min timeframe, which varied with MW and skin layer. Using multiphoton microscopy, image analysis and a Fick’s law analysis with 2D cartesian and axisymmetric cylindrical coordinates, we reported experimental trends of epidermal and dermal diffusivity values ranging from 1–8 µm^2^ s^−1^ to 1–20 µm^2^ s^−1^ respectively, with a significant decrease in the dermal-epidermal junction of 0.7–3 µm^2^ s^−1^. In breaching the stratum corneum (SC) and dermal-epidermal junction barriers, we have demonstrated practical application, delivery and targeting of macromolecules to both epidermal and dermal antigen presenting cells, providing a sound knowledge base for future development of skin-targeting clinical technologies in humans.

## Introduction

The skin has long been employed as a route for the delivery of bioactive molecules for local and systemic effects, and more recently, a route for the detection of analytes^1^, with the particular advantage of avoiding hepatic first-pass metabolism. The stratum corneum (SC) is considered to be the main barrier to penetration of actives^2^ and the removal of analytes. The underlying strata, the viable epidermis (VE) and the dermis are often the main targets of the compounds delivered through the skin for local and systemic delivery, as well as the detection of circulatory biomarkers. Whilst there are now a number of topical and transdermal products on the market^3^, there are many actives which do not readily permeate the SC. Much research in the field of skin delivery has now been devoted to the development of strategies to overcome the SC barrier and enhance topical and transdermal penetration for poorly permeating actives. The principal solutions to the enhancement of percutaneous absorption involve chemical methods, such as the use of penetration enhancers, and physical methods such as iontophoresis, sonophoresis and electroporation^4–7^.

In the past several decades, there has been considerable interest in the development of nanotechnologies for improved topical and transdermal delivery. These include formulation-based nanosystems, such as colloidal nanocarrier systems (nanoemulsions, solid lipid nanoparticles (SLNs), nanostructured lipid carriers (NLCs)), flexible nanovesicles: elastic liposomes, transfersomes, ethosomes and niosomes, and microdevices involving microneedle-based technologies^8–11^. The latter physically and mechanically overcomes the SC to deliver actives directly to the VE and dermis. A wide range of pharmaceutical formulations, with varied purposes and molecular weights (MWs), have been delivered using microneedles, including naltrexone^12,13^, verapamil, amlodipine^14^, plasmid DNA^15^, cosmeceutical peptides (melanostatin, rigin and pal-KTTKS)^16^, insulin^17,18^ and macromolecules such as human growth hormone^19^ and vaccines^20–23^. Microneedle, skin-targeted devices have also been used to extract biomarkers for disease diagnostic applications in influenza^24^ and dengue^25^, providing a new and rapid way of detection without the need to draw blood from patients.

Vaccines are now mainly given by injection into intramuscular and subcutaneous tissues. The skin provides an ideal alternative and shallower delivery target as it is capable of producing an effective immune response mediated by antigen presenting cells (APCs) found in the Langerhans cells in the VE and dendritic cells and macrophages in the dermis^26^. In contrast, needles and syringes lack the precision to target the APCs that are within the micrometre range from the surface of the skin, instead delivering vaccines into immunologically-poor regions of the body (i.e. muscle). We have recently demonstrated that targeting the skin’s immune system with the Nanopatch technology produced a more efficient immune response with 1/100^th^ of the dose usually administered intramuscularly, and also avoided the pain and discomfort associated with traditional methods of vaccine delivery^27,28^.

Given the enormous potential^29^ of nano-delivered vaccines in both the developed and developing world, namely vaccine thermostability, reduced costs and elimination of sharps injury and possible self-administration, it is imperative to understand the diffusion of the macromolecules in the skin *after* administration to predict their availability for uptake by cells in proximity. This will also provide sound knowledge on the ability to extract analytes from the skin, for them to diffuse near capture surfaces. We have shown that solute diffusion is dependent on MW and differs in the individual layers of the skin, which possess distinct mechanical and structural properties for both small solutes^30^; local deep tissue penetration of compounds after dermal application: structure-tissue penetration relationships^30^; molecular size as the main determinant of solute maximum flux across skin^31^. and, in mice, macromolecules, where diffusion gradually increased as the depth from the skin surface increased^32^. We have reported significant differences between mice and human skin in small solute permeability^33^ and in the stiffness of the skin^34^, it is expected the diffusion kinetics of macromolecules in the VE and dermis would also vary between the two species.

The primary barrier to effective skin delivery of solutes is the highly-ordered lipid-rich region of the Stratum Corneum (SC). Microneedle devices overcome this barrier by piercing the SC to enable direct deposition of their cargo and its extraction within the epidermis and the dermis. However, for effective delivery, this deposited vaccine/drug or extracted biomarkers must diffuse through the surrounding tissues to reach their target sites.

The permeability of various solutes have been investigated in various animal skin models, such as rodent^32^ and porcine skin^35^, as potential surrogates for human skin and shown to possess different permeabilities^33,36^, skin structures, composition and mechanical behaviour^34^ from human skin. However, how macromolecule solute diffusivity in each layer relates to the underlying skin morphology is not well defined and this knowledge is of importance in further developing various human skin delivery devices using animal skin findings.

Several studies have reported a range of diffusion coefficients for macromolecules in animal and human skin. Raphael *et al*. studied the diffusion of 70 kDa and 2 MDa RD in mouse skin, and reported diffusion coefficients highly dependent on skin layers, in^32^. Römgens *et al*. reported a similar diffusivity for 500 kDa fluorescein labelled dextran in the epidermis (8 µm^2^ s^−1^) in the dermis (9 µm^2^ s^−1^) and a somewhat higher diffusivity (23 µm^2^ s^−1^) for a 40 kDa macromolecule diffusion in human skin using fluorescence recovery after photobleaching (FRAP)^36^. Mansoor *et al*. reported that microneedle delivery of doxorubicin (~580 Da) in pig dermis was associated with a doxorubicin diffusivity of approximately 0.5–4.2 µm^2^ s^−1^^35^.

**Figure 1:**
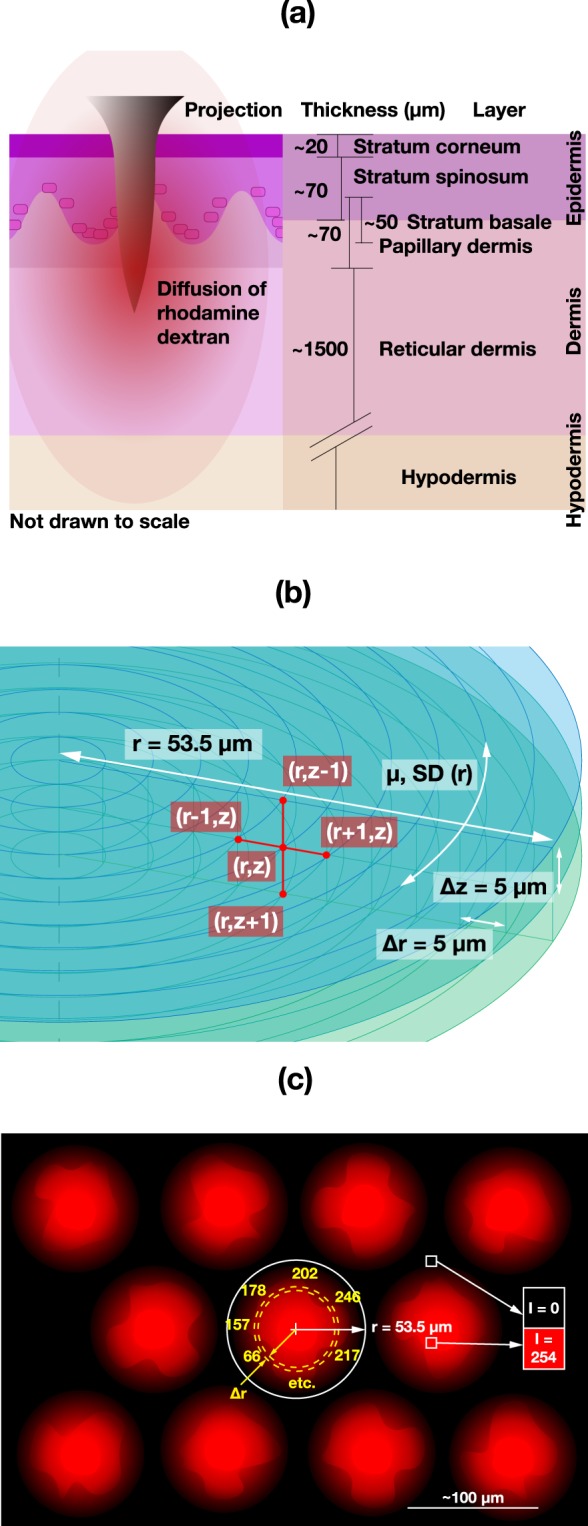
(**a**) Schematic illustrating one Nanopatch microprojection applied into the skin with the diffusion of RD within the different layers of human skin. (**b**) Allocation of coordinates (three z-planes shown) for analysis, where RD intensities are recorded along each circumference of 5 µm, in radial increments up to the midpoint between two projections, with a depth spacing of 5 µm as well, from the skin surface down to 100 µm. (**c**) A schematic example of extracted intensity values from the centre of puncture hole with SD in one layer and at one time point.

Here, we investigated the diffusion properties of macromolecules in every stratum of the human skin (see schematic Fig. 1(a)) after the application of a Nanopatch, an ultra-high density microneedle array vaccine delivery patch. This included the dermal-epidermal junction, a region that hinders diffusion of both lipophilic and hydrophilic compounds into the dermis. The diffusion resistance is due to the tight adherence of stratum basale (SB) cells through tight junctions, desmosomes and linker proteins, and the densely packed dermal collagen invaginations of papillary dermal cells^37,38^, that act as a physical barrier for large molecules^39^. This contrasts with the loosely arranged collagen matrix found in the deeper dermis that provides less structural resistance than the epidermis to diffusion to target cells within the region. In this study, we disrupted both the SC and dermal-epidermal barriers physically for macromolecule delivery.

Typical sizes of vaccines can range from ~14 kDa^40^, ~70 kDa^41^ to ~100 kDa^42^, and even in the megadalton range for DNA plasmid vaccines^43^ with 1000 s of base pairs (bp), with one bp ~650 Da. For example, influenza vaccine proteins, namely haemagglutinin and neuraminidase, have a MW of ~77 kDa and ~60 kDa respectively^44^ although these proteins typically exist as polymers, weighing ~230–240 kDa, with some reported to be in the order of ~670 kDa^45^. We used rhodamine-labelled dextrans (RD) as a model for large molecules being delivered into the skin. We selected 70, 500 and 2000 kDa to represent the low, intermediate and high vaccine MWs. The RDs were delivered into *ex vivo* human skin with the Nanopatch and their diffusion was imaged over time using high-resolution multiphoton microscopy (MPM). The acquired images were then processed to calculate the diffusivity of these macromolecules in every imaged skin strata. This understanding of the diffusion properties of relevant molecular sizes in human skin will be crucial for vaccine and drug delivery in translational clinical research.

## Results

In this paper, we have first characterised the Nanopatch microprojections used in the experiments shown in Section 2.1. We then used a maximum and minimum threshold analysis to provide a visual illustration on where the dye was found in space and time. The maximum and minimum concentrations of the diffusion fronts show how they reduce and clear out over time, presented in Section 2.2. Finally, the third major results of this work in Section 2.3 presents the diffusion coefficient analysed with Fick’s second law (enabling comparison with many diffusion studies), which interprets the subtle differences between the neighbouring (in both space and time) pixel values, rather than at the two extreme thresholds. In this case, we used the coefficient to highlight the local reduction in the dermal-epidermal junction region.

### Penetration of the Nanopatch into *ex vivo* human skin

**Figure 2:**
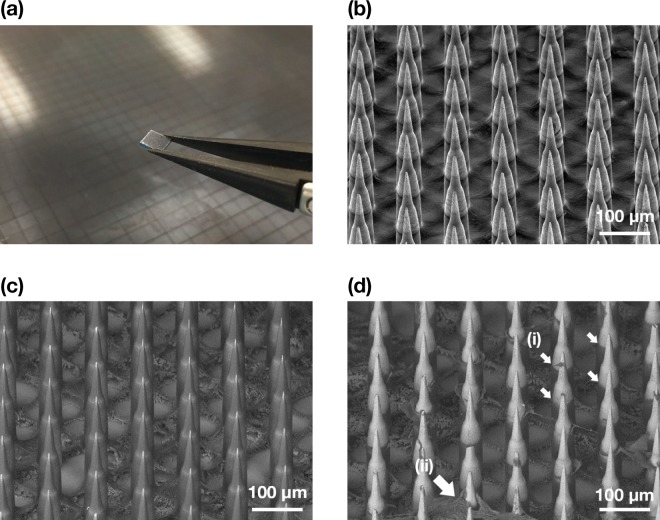
(**a**) Photograph of a 4 × 4 mm Nanopatch held with tweezers in front of a Nanopatch wafer. (**b**) Representative image of secondary electron (SE) detector SEM of uncoated Nanopatch projections at a 45° tilt. (**c**) Representative image of backscatter electron (BSE) detector SEM of a coated Nanopatch. (**d**) Representative BSE detector SEM of a coated Nanopatch applied on full thickness *ex vivo* human skin and removed. (i) Dissolved coating representing the extent of skin penetration, and (ii) skin fragments lodged on microprojections are indicated as shown. Images shown in (**a**–**d**) are different patches but from the same wafer.

**Figure 3:**
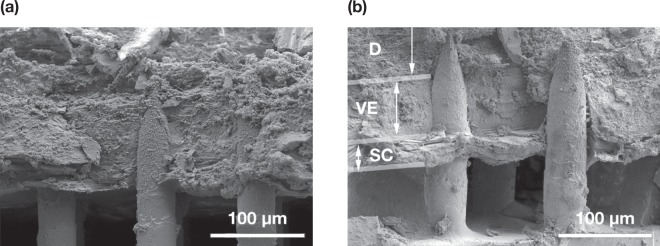
(**a**,**b**) Representative Cryo-SEM images of transverse-fractured *ex vivo* human skin with Nanopatch applied to the skin. The microprojection, stratum corneum (SC), viable epidermis (VE) and dermis (D) were labelled on (**b**).

Figure 2 illustrates an SEM image of a Nanopatch prototype diced into a 4 × 4 mm square (a) and the uncoated projections in (b), and coated projections prior to skin administration (c) and removed approximately halfway down the projection shafts after administration (d). This demonstrates the deposition of the coating formulation from the projections to the skin. Several corneocytes detached from the SC were also observed stuck onto the microprojections as indicated by the arrows. Penetration depths of the microprojections into full thickness skin and dermatomed dermis were measured at least 100 times as 149 ± 49 µm and 128 ± 40 µm respectively. The amount of formulation deposited into the skin was quantified as 30.6 ± 5.05% of total amount coated onto the microprojections. Figure 3 displays cryo-SEM images of patched skin with the Nanopatch remaining in the skin. The SC, VE and dermis were distinguished, as indicated in (b).

### Assessment of rhodamine dextran diffusion and clearance

**Figure 4:**
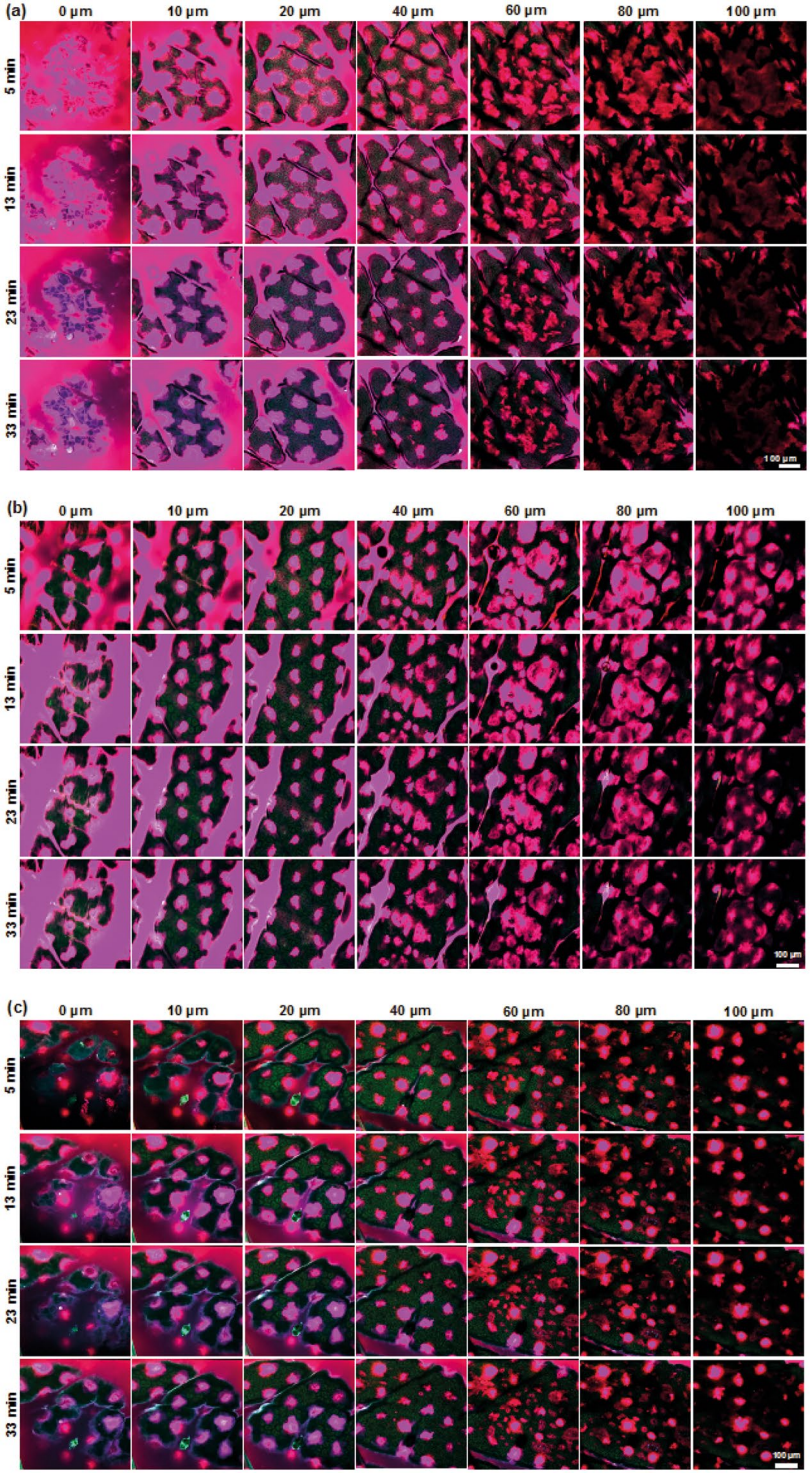
Representative multiphoton images of *ex vivo* human skin at different depths at 5, 13, 23 and 33 minutes post Nanopatch application onto the skin. The Nanopatch was coated with (**a**) 70 kDa (**b**) 500 kDa and (**c**) 2000 kDa rhodamine-labelled dextran, applied onto the skin using a spring-loaded applicator and left on the skin for 2 minutes to allow sufficient time for coating dissolution and approximately 3 minutes for excision and loading of the specimen onto the microscope stage and initialising it before the first images were captured. All images were pseudocoloured to highlight rhodamine-dextran (magenta) and skin (green) emissions.

Representative images of the patched skin at selected time points and depths are displayed in Fig. 4(a–c), demonstrating the successful delivery of 70, 500, 2000 kDa RD to a minimum depth of 100 µm in human skin *ex vivo*. Each RD MW deposited showed similar diffusion patterns. Significant deposition of all RD coatings was observed on the skin surface. Some coating was also collected in the furrows, as shown in the images of the VE (10–40 µm in depth). The patched areas were clearly identifiable as circular, regular spots in each stratum. A complete representative stack, inclusive of all time points and depths in videos is available in the Supplementary Figs S1–S4.

**Figure 5:**
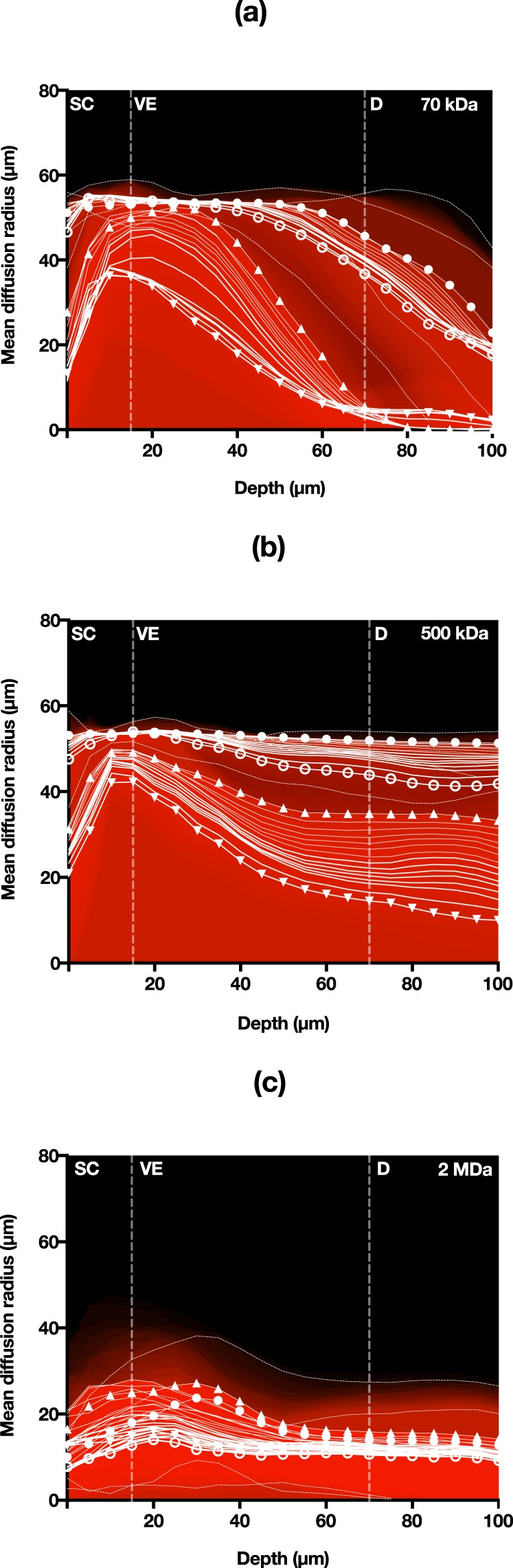
Mean RD clearance rate detected at 5% and 95% threshold for (**a**) 70 kDa, (**b**) 500 kDa and (**c**) 2 MDa. ▲ = 5% threshold – 5 mins, ▼ = 5% threshold – 33 mins, ● = 95% threshold – 5 mins, ○ = 95% threshold – 33 mins.

Upon closer observation of Fig. 4, diffusion fronts were separated into two distinct regions from the centre of the microprojection holes. An intense and fully saturated region that was separable for analysis as a distinct area when a 5% threshold was employed, corresponds to a very high concentration of RD, likely was in direct contact or very close to microprojections during the initial payload deposition. We also observed a larger, outer region, captured with a 95% threshold, corresponding to the minimum detectable fluorescence diffusion front. This was denoted as the farthest distance the molecule has spread. Release profiles (diffusion fronts) of both maximum and minimum saturation for 70, 500 and 2000 kDa in human skin *ex vivo* over 30 minutes (Fig. 5), showed that the molecules dissipated (i.e. mean diffusion front radius decreased) over time at each location. This represented molecular diffusion of concentrations to below detectable thresholds. In terms of depth, all RDs appeared to increase in the spread from the surface, peaking at approximately the SC-VE boundary.

The rate of dissipation was quantified using Fig. 5 as the mean change in radius over time for each skin layer (Table 1) using the maximum intensity threshold radius observed. The times show an estimation of the mean duration for each layer the radius would reduce to zero. These values were found to be proportional to the MW and increased from the SC to VE. However, a reduction in the clearance rate was estimated for regions towards the dermis. No clearance information was available for 70 kDa RD at deep layers, likely due to rapid clearance of diffusion fronts prior to imaging, in contrast to stagnation of 2000 kDa RD.

**Table 1:** Time for fully saturated RD (5% threshold) to clear out in each skin layer from a microprojection site estimated using Fig. 5.

Layer/MW (kDa)	Time (min)		
	70	500	2000
Stratum corneum	42	61	65
Viable epidermis	31	37	67
Dermis	n/a	25	124

### Experimentally derived diffusivity values by intensity profiling

**Figure 6:**
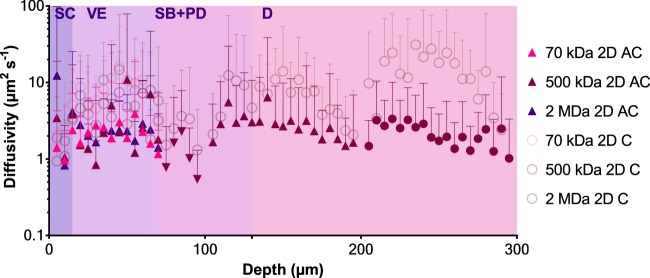
Diffusivity of RD in *ex vivo* human skin for 70 kDa, 500 kDa and 2 MDa. Curve fits are not continuous due to the analysis of separate stacks and truncation of layers lacking signal. Four replicates per molecule size. Error bars indicate SD. SC = stratum corneum, VE = viable epidermis, SB + PD = stratum basale and papillary dermis junction, D = dermis. AC = axisymmetric cylindrical, C = cartesian.

Diffusion data was calculated as a quantifiable value by comparing intensities of each pixel along multiple concentric radii from the projection centre (Fig. 6). An inverse proportionality between MW and diffusivity can be observed. The 2 MDa RD appeared to have slightly higher diffusivity values than 500 kDa, although within error. All three MWs demonstrated diffusivity coefficients in the same order of magnitude, and because of this, the deeper skin layers were subsequently investigated directly using the 500 kDa RD. Two models are presented – in 2D cartesian and axisymmetric cylindrical coordinates. Both results are closely related to each other, although the cartesian coordinate modelling appeared to have continually increasing diffusivity in the dermis, in contrast to the cylindrical model, which appeared flatter. Nevertheless, both models illustrate a local reduction in the dermal-epidermal junction region. The SC diffusivity of the three MWs was ~1–5 µm^2^ s^−1^. Diffusivity doubled from the SC to the VE, to approximately ~2–10 µm^2^ s^−1^ in the case of the cartesian model, and slightly less at ~2–3 µm^2^ s^−1^ in the cylindrical model. Both reduced again to the lowest values in the dermal-epidermal junction at ~0.7–3 µm^2^ s^−1^. Diffusivity increased again past the dermal-epidermal junction and continued to rise to the highest point in the reticular dermis at an average of 10–20 µm^2^ s^−1^, and remained at approximately 1–3 µm^2^ s^−1^ in the cylindrical model.

## Discussion

In this work, we have shown direct deposition of fluorescence-labelled, macromolecules with a range of MWs into the epidermal and dermal regions of human skin, overcoming the barrier effects of the SC and the dermal-epidermal junction by using a Nanopatch device (microneedle array drug delivery patch). The microprojections of the Nanopatch physically disrupted the SC and the dermal-epidermal junction to overcome these barriers to epidermal and dermal delivery. Macromolecules were then delivered to the viable epidermis and the dermis, where APCs critical for immune response reside (i.e. VE – Langerhans cells; dermis – dermal dendritic cells)^27^. In comparison, needles used for intradermal injections are too large to target APCs accurately. The subsequent macromolecule diffusion within the skin strata demonstrated, quantified and modelled allows the prediction of transport to target sites within the skin, including to the microcirculation for transdermal delivery. The data was collected using non-invasive MPM from all layers of skin and analysed using efficient yet straightforward modelling tools. Apart from a few *in vivo* studies^46^, our work, using *ex vivo* human skin and its application delivery method, is the closest model representing clinical and real-life conditions, which is highly valuable in demonstrating the potential for novel skin-based vaccine delivery. This work will inform the development of a wide range of nano- and micro-technology based skin delivery systems for macromolecules.

The SC is widely accepted to be the primary barrier to penetration of any topically applied compound larger than 500 Da to the skin^47^. We have identified first that the dermal-epidermal junction provides a second barrier to diffusion of molecules both in the drug and vaccine size range. The decreased diffusivity values measured at the two barriers (SC and dermal-epidermal junction), relative to the VE and dermis (approximately one order of magnitude difference) is likely associated with the unique morphology and organisation of the barrier regions^34^. We suggest that the dermal-epidermal junction may be as formidable a barrier as the SC, as demonstrated by the reduction in lateral diffusion, even with microprojection intervention (Fig. 6), which is significantly higher than topically-applied formulations of similarly sized molecules^32^. We also believe that the dermal-epidermal junction is likely to inhibit vertical diffusion of the RDs further. This supports the previous findings of Andrews *et al*. proposed that the entire epidermis could limit transdermal delivery^48^. This is important, as the focus of the design of skin delivery systems for the past 40 years has been to overcome the SC, without the acknowledgement of this second barrier region.

Microprojection-based delivery systems provide unique advantages in transdermal delivery, as they breach the SC, epidermis and the dermal-epidermal junction. Here, the Nanopatch was placed in the skin for two minutes, an acceptable and representative administration protocol of a clinical scenario for immunisation delivery. We expect diffusivity to be even higher in the presence of blood circulation in an *in vivo* model, due to increased movement of inter- and intracellular fluids; and we, therefore, speculate a further reduction of delivery timeframe compared to the mouse model^32^, while achieving similar delivery efficiencies. We also expect diffusivity to be approximately constant throughout the reticular dermis in the current *ex vivo* condition, given that its structure remains mostly homogenous through its full thickness, but it may be dependent on the addition of blood flow in the *in vivo* state^49,50^.

We also demonstrated clearance of RD from the skin layers, that had not been observed in the mouse model^32^. It is possible that the Nanopatch configured with larger microprojection geometry (250 µm *versus* 100 µm) and lower microprojection density (10,000 cm^−1^ *versus* 20,000 cm^−1^) in this study allowed sufficient amounts of RD to dissipate into the greater tissue volume, hence our observation of the diffusion fronts dissipating rather than spreading outwards from projection centres. This efflux diffused to concentrations below the detectable threshold over time. It is unlikely that the RD diffused against the concentration gradient to fully saturated projection centres (Fig. 5). This’dissipation’ effect was also observed by Mansoor *et al*. investigating doxorubicin concentrations in the VE of porcine skin models, a skin type that has more structural similarity to human skin in terms of skin layer thicknesses^35^ than mouse skin.

Although the diffusivity of 70 kDa RD was slightly higher than 500 and 2000 kDa RD (Fig. 6), they were well within the SD in the SC and VE (~1–5 µm^2^ s^−1^ and ~2–10 µm^2^ s^−1^ respectively). A marked reduction of diffusivity (~0.7–3 µm^2^ s^−1^) was observed in the dermal-epidermal junction. Diffusivity of 500 kDa RD was highest in the dermis (10–20 µm^2^ s^−1^, 2D cartesian model only). Regardless of model, these values highlight the importance of characterising diffusivity in each skin strata separately due to the dependency along the depth of the skin, cf. literature explored earlier reporting simplified or singular diffusion coefficients, suggesting a close correlation between the ability of the compound to diffuse and the skin’s physical and mechanical characteristics (e.g. proportional to elasticity, and an inversely proportional to viscoelasticity)^51^. Secondly, provided in this paper are two models – 2D cartesian and axisymmetric cylindrical coordinates. The former was provided enabling direct comparison with Raphael *et al*.’s work^32^ illustrating a very similar rise-and-fall trend along the depth of the skin. However, an improvement to the accuracy of the modelling using cylindrical coordinates accounted for the expanding volume as the fluorescence progress farther outwards from the axis centre, saw discernible differences compared to the former model. While we believe this may be more accurate to analysis diffusion from microprojections, it does not provide definitive diffusion coefficients of the human skin. Nevertheless, it does, however, suggest that our results are sufficiently accurate, considering the inter- and intra-specimen variability in biological tissues.

While not explored in the scope, the partition coefficient *P*, measuring hydrophobicity or lypophilicity as *log P* may be as important as molecular weight. The hydrophobicity would depend on the formulation constituents: for example, hydrophilic: proteins, peptides, nucleic acids, carbohydrates, haptens, and hydrophobic: lipopeptides, antigens, adjuvants, linker molecules^52^. Given that cells and intercellular spaces are well-hydrated in the viable skin layers, a more hydrophilic formulation may exhibit higher diffusion rates than a hydrophobic formulation – and this should be explored as future work by applying the same diffusion model proposed here.

Our results appear to agree with most other studies in the literature as discussed earlier, although it is interesting to note that the diffusion coefficients calculated in this work, in human skin, are approximately one order of magnitude higher than in mouse skin. This difference is most likely being determined by corresponding differences in the different skins’ physical and mechanical properties. For instance, the mouse skin is more viscoelastic than human skin, with different cellular structures impeding diffusion^34^. In contrast, the more porous structure associated with the dermal collagen network of human skin would be expected to facilitate macromolecular diffusion in the dermis, relative to that in the packed collagen matrix of mouse skin^34^. These findings highlight the importance of using human tissues in defining diffusion related to developing devices for human skin delivery of macromolecules, and as a minimum, a need to correct for skin structural differences when predicting their effects on skin permeation using animal models.

Overall, Nanopatch-delivered macromolecules were shown to diffuse through skin layers, including the permeation barriers of the SC and dermal-epidermal junction. Diffusivity values for the range of MWs obtained within this work is relevant for informing the development of skin-targeting medical devices delivering vaccines and drug molecules. Differences in skin diffusivity between the present work in human skin and past work in mouse skin clearly exist. The findings suggest there is a need for more literature data based on similar experimental methodologies to assist in both the development and the translation of these device technologies into clinical use.

## Conclusions

Understanding and quantifying the diffusivity of macromolecules in the skin underpins the development of novel, microscale devices that effectively target skin delivery. We investigated macromolecule diffusion in all layers of *ex vivo* human skin through the minimally invasive application microprojection arrays to deposit RDs of various MWs, and imaged the skin using non-invasive MPM. We demonstrated a rapid clearance within 30 minutes in the skin of relatively small RDs (70 kDa), and stagnation of larger molecules (2000 kDa). Diffusivity values increased from the skin surface (~3–8 µm^2^ s^−1^) down to the papillary dermis (~1–20 µm^2^ s^−1^, depending on the model) but a significant decrease in diffusivity was observed in the dermal-epidermal junction (~0.7–3 µm^2^ s^−1^), which would have impeded diffusion through to the dermis without direct breaching and deposition by the microprojections. We propose that our human skin model is the closest to *in vivo* conditions. Indeed, our minimally invasive medical device coupled with our non-invasive characterisation techniques could inform drug diffusivity in individuals, and permit the tailoring of nano-delivery systems for personalised medicine in the future. Quantifying the diffusivity properties of macromolecules for a range of MWs in human skin down to the individual skin stratum could provide a significant step in more accurate design and development of the fabrication, geometry and drug formulations used in these technologies.

## Materials and Methods

### Ethics statement

All work carried out has been approved by The University of Queensland Human Research Ethics Committee (approval number 2008001342). Written informed consent was obtained from all participants. All experiments were carried out in accordance with The University of Queensland guidelines.

### Nanopatch manufacture

Solid microneedle arrays (Nanopatch) were manufactured from silicon wafers using the Deep Reactive-Ion Etching method^53^ at the Australian National Fabrication Facility – Queensland Node (ANFF-Q) (Brisbane, Australia) to a specification of 10,000 projections per cm2, tapered to approximately 250 µm in length and 40 µm base width, tapering to a cone tip of ~15°. Wafers were diced into 4 × 4 mm squares for coating and application. Nanopatch patches tilted to 45° were examined with a scanning electron microscope (SEM) (SU3500, Hitachi, Tokyo, Japan and JCM-5000, Jeol, Tokyo, Japan) with secondary electron detector (uncoated) or backscattered detector (coated/applied) at 10–15 kV.

### Nanopatch coating

70, 500 and 2000 kDa rhodamine-labelled dextrans were chosen as models of low, medium and high molecular weight (MW) macromolecules. The coating formulation was similar as per previously^54^ (for penetration depth investigation) except for replacing the FluoSpheres component with ^14^C (for delivery efficiency investigation), or RD of one MW per patch (for diffusion investigation). Patches were air-dried using jet-assisted airflow as per Chen *et al*.^55^.

### Skin tissue collection, preparation and characterisation

Excess abdominal skin was collected from female patients undergoing abdominoplasty (*n* = 15 donors, *m* = 36 years old, *SD* = 7.8). Subcutaneous fat was discarded, and the skin was wrapped in aluminium foil and stored at 4 °C until the commencement of the experiment (<48 h), or frozen at −20 °C and sealed airtight (>48 h). Dermatomed dermis was also prepared by removing 200 µm from the skin’s surface (Padgett Model B, Integra Lifesciences, NJ, USA). Skin specimens were allowed to return to room temperature (~22 °C) before the Nanopatch application. Skin used in this study was characterised previously^34^ for its physical and mechanical properties.

### Experimental design

Experiments to study RD diffusivity in the SC, VE and superficial papillary dermis were carried out on fresh full thickness human skin, imaged from the surface to a depth of 100 µm. Four replicates were carried out per RD MW. Following this, we investigated diffusion in the dermal-epidermal junction, the rete and ridges region by applying the Nanopatch directly on to the dermis (full-thickness skin with epidermis removed ~200 µm from the surface with a dermatome) and imaging from the new surface to 100 µm. Three replicates were carried out with 500 kDa RD. Subsequently, lateral diffusion of 500 kDa RD in the dermis was studied by application of the Nanopatch on three skin replicates of thawed full-thickness skin. Past studies using carefully-handled frozen skin demonstrated suitability for drug diffusion^16,56^. Images were taken from a depth of 100–200 µm.

### Nanopatch application onto skin

A simplified diagram illustrating the skin strata and microprojection penetration is shown in Fig. 1(a). Experiments were carried out at the Translational Research Institute (Woolloongabba QLD, Australia). To apply a Nanopatch, the skin was first placed on ten plies of 0.9% w/v saline-moistened (Baxter, NSW, Australia) folded Scott Compact Towels (Kimberley-Clark Australia, Milsons Point NSW, Australia) in a polystyrene tray (similar to Jee *et al*.) maintaining pH and osmolarity)^57^. The skin was held down prior to application to mimic *in vivo/in situ* tension. An application velocity of ~15 m s^−1^ was achieved with an in-house developed spring-loaded applicator. The patch was removed two minutes post-application to allow sufficient time for the coating to dissolve^54^. Patched skin area was immediately excised and imaged. The first imaged time point is approximately five minutes after the moment of patch application when the excision of skin and the search for a suitable area and microscope configuration were factored in.

### Measurement of microprojection penetration depth and delivery efficiency

Microprojection penetration depth was measured using histology sectioning, as detailed by Crichton *et al*.^54^ Penetration tracks shown with delivered FluoSpheres were measured from the surface of the skin as the penetration depth. At least 100 tracks in total were measured *(n* = 4). Delivery efficiency was used to measure the amount of vaccine eluded into skin *versus* amount remaining on the patch. The method was identical as per previous studies^28^. In brief, patches were coated with ^14^C-labelled ovalbumin (American Radiolabeled Chemicals, MO, USA) with 3–4 replicates applied onto four skin samples each.

### Cryo-SEM imaging

Representative images illustrating Nanopatch application through the skin surface were captured using Cryo-SEM (XL30, Philips, Amsterdam, Netherlands; JSM-7100F, JEOL, Tokyo, Japan). As previously^27^ and similarly^58^, with exceptions in brief: the Nanopatch was applied onto skin as described in Section 5.6, but excised with the patch *in situ* and adhered (Tissue-Tek OCT Compound, Sakura Finetek, Alphen aan den Rijn, Netherlands) onto the specimen stage. The patch remained on the skin (cross-sectional view) and fractured with utility pliers in liquid nitrogen or was removed (top-down view) prior to submerging. The sample was then sealed and transferred to the cryotransfer chamber under vacuum to prevent moisture contamination. Samples were iridium coated for two minutes prior to imaging and sublimated when applicable. Temperatures were set to −145 °C in the chamber and pre-chamber; −195 °C for the anti-contaminator and −105 °C for sublimation (over ten minutes). Images were captured using secondary electron detector, accelerating voltage of 2–5 kV. Images obtained were not post-processed except for brightness and contrast adjustment, and labelling.

### Multiphoton fluorescence imaging

Multiphoton fluorescence images of patched skin were acquired using the LaVision Biotec Nikon Ti-U inverted microscope (Bielefeld, Germany) equipped with a 20 × 0.95 NA water immersion lens (Olympus, Tokyo, Japan) and Titanium:Sapphire femtosecond-pulse Spectra Physics Mai-Tai laser (Santa Clara CA, USA). The excitation wavelength was set at 760 nm. A stack of 500 × 500 µm images with a pixel resolution of 1047 × 1047, along the Z-plane with a step size of 5 µm was obtained. Imaging power when imaged for depths between 0–100 µm was set to 14.5 mW and increased to 47 mW for depths of 100–200 mW. Each replicate was imaged for 30 minutes.

Emitted light was separated into three channels and detected by their respective bandpass filters (410–485 nm (cellular auto-fluorescence); 485–550 nm (cellular auto-fluorescence and RD); and 550–670 nm (RD)), with one raw image stack generated per emission channel.

Multiphoton images were processed using Fiji^59^. The emission channels were pseudo-coloured green (λ_ex_: 410–485 nm), blue (λ_ex_: 485–550 nm) and red (λ_ex_: 550–670 nm) and merged to form a single image stack and saved as.jpg images. Images from the green and red channels were saved separately in.jpg format. Space-time reconstruction images and videos were created using Imaris 6.3.1 (Bitplane, Belfast, United Kingdom).

### Diffusion analysis

Images obtained from the 550–670 nm filter as described in Section 5.9 were first assessed qualitatively, then in Matlab R2016b/R2017a (MathWorks, MA, USA) for quantitative profiling – measurement of the distance of RD diffusing from the centre of the hole and the associated fluorescent intensities. All replicates were analysed individually as a single stack, containing the entire z-plane, then repeated for all time points. Between four to five visible projection holes in all layers, away from flooded furrows, were selected. The same coordinates were also used to characterise the puncture hole aperture in images obtained from the 410–485 nm filter. The centre of the projection hole was selected manually for all analyses. A circular area with a diameter of 107 µm (corresponding to equidistant spacing between projections) was chosen at the centre of each projection hole for diffusion analysis, and a diameter of 50 µm (slightly larger than projection size) was used for analysis of hole aperture to account for any relative changes in hole size over time. The centre of the area was chosen manually.

Two methods were developed to quantify the diffusion: a visual analysis approach looking at the maximum and minimum diffusion fronts of the dye in space and time to estimate clearance in Section 5.10.1 and determining the diffusivity values by comparing neighbouring pixel changes in space and time using Fick’s second law of diffusion in Section 5.10.3.

#### Assessment of the rate of spread from puncture hole centres

**Figure 7:**
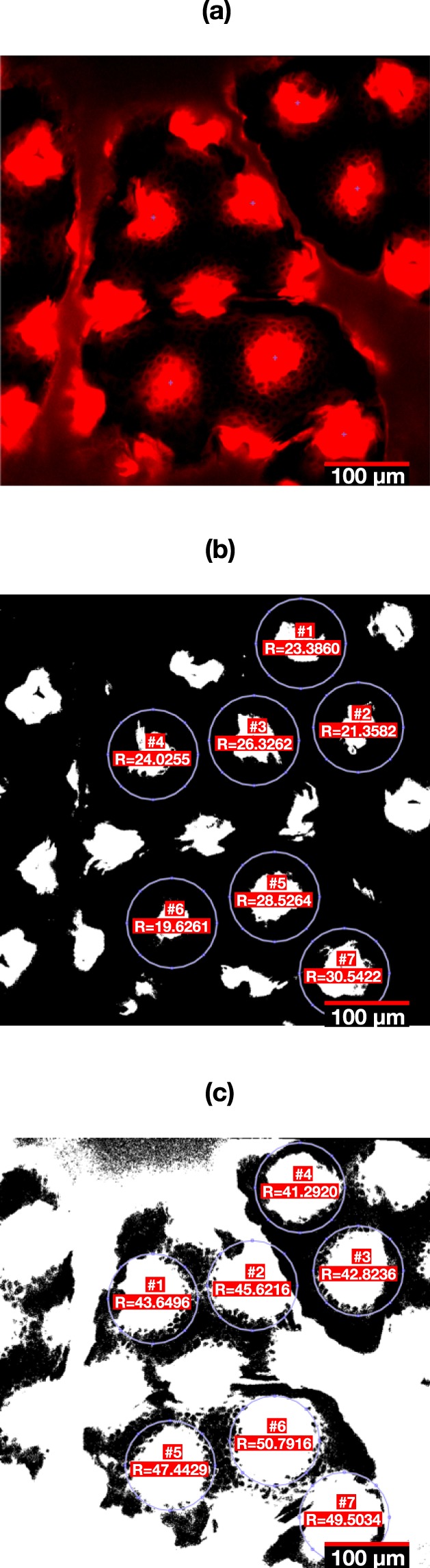
Method of estimating mean diffusion radius using fluorescence thresholding to detect maximum and minimum detectable spread of RD. (**a**) Representative image of patched skin, pseudocoloured to represent RD. (**b**) Fluorescence threshold at 5%. (**c**) Fluorescence threshold 95%. Three to four puncture holes were selected for analysis; shown here are seven holes for demonstration purposes. Overlay graphics generated from Matlab code directly.

This is a quantitative method to determine the extent of the spread of RD within the skin. We calculated the mean distance (radius) from the puncture hole centre by applying two cut-off filters at 5% and 95% brightness threshold to quantify the spread at its maximum saturated intensity and the minimum detectable intensity, respectively, using the Matlab function im2bw. These two cut-off values were selected to account for some of the background signal (noise) captured during the imaging process. Two cut-off values were used, as the initial assessment of the images showed a small region at the centre of projections where the intensity is fully saturated, and an outer, surrounding region where RD signal was less intense. The ratio between the threshold region and the circle area (Fig. 7(b,c)) was used to calculate the mean radius from where the molecule has spread. This information is useful because it indicates the distance travelled and the concentration of RD in the skin over time. The rate of spread incorporates changes (if any) in projection hole size over time from the auto-fluorescence channel. Maximum intensity indicates the zones with the highest concentration of RD, which is from where the molecule *should* diffuse. Minimum intensity provides information on the farthest distance to which the particle has dispersed, irrespective of concentration. The mean rate of spread of the maximum concentration threshold was used to estimate the time to complete clearance, when the mean radius in each layer reduces to zero. This data is presented in Table 1.

For this analysis, images were first converted to greyscale using Matlab function rgb2gray, and the intensity was normalised using the function imadjust and binarised with the function im2bw. Mean radius was then calculated using the ratio between the detected area in white and the area of the full circle. A representative example is shown in Fig. 7. The results of all selected projections were averaged with standard deviation recorded. Results were plotted and analysed in GraphPad Prism 7 (GraphPad Software, CA, USA).

#### Extraction of intensity values for diffusion analysis

Here, we analyse the images for the diffusion coefficient analytically with Fick’s second law by first determining the intensity of each pixel from the projection centres. Similar to the previous section, the images were converted to greyscale, and their intensities were normalised. Fluorescence intensity was assumed approximately proportional to concentration as proposed by previous bodies of work, e.g. Raphael *et al*.^25^ Dada *et al*.^60^ and Mohammed *et al*.^16^. Brightness intensities (0–255) were captured circumferentially to the nearest pixel and concentrically at every 5 µm from the centre up to the midpoint between two projections, analysing at the same coordinates over depth (skin surface down to 100 µm also at 5 µm spacing) and time (immediately after patching for 30 mins at 2 mins spacing). Results of all selected projections were averaged with standard deviation exported.

#### Modelling of diffusivity with Fick’s second law

Intensity values from the previous section were used to calculate diffusion coefficients using Fick’s second law of diffusion by quantifying the change in RD intensity at each location over time (for a one-dimensional diffusion analysis):

1 $$\frac{\partial C}{\partial t}=D\frac{{\partial }^{2}C}{\partial {x}^{2}}$$

where *C*(*x*, *t*) is the concentration of RD and is dependent on position. The intensity was first averaged along each circumference assuming axisymmetric spread from the projection hole centre (no dependence on the angle *θ*). Rearranging for the diffusion coefficient *D* and modifying for 2D Cartesian coordinates, similar to Raphael *et al*.^25^, yields the following equation:

2 $$D(r,z,t,\partial t)=\frac{\frac{{\partial }_{t}C}{\partial t}}{\frac{{\partial }_{r}^{2}C}{\partial {r}^{2}}+\frac{{\partial }_{z}^{2}C}{\partial {z}^{2}}}$$

where *r*, *z*, *t*, *∂t* refer to: radius from projection hole centre, depth from the skin surface, time and time step respectively. A further analysis of the same data using a more refined, 2D axisymmetric cylindrical coordinate system, to account for the expanding volume as the analysis moves away from the axis centre^61^.

3 $$D(r,z,t,\partial t)=\frac{\frac{{\partial }_{t}C}{\partial t}}{\frac{{\partial }_{r}^{2}C}{\partial {r}^{2}}+\frac{1}{r}\cdot \frac{dC}{dr}+\frac{{\partial }_{z}^{2}C}{\partial {z}^{2}}}$$

The results from the two analyses are shown in Fig. 6. Assuming neighbouring pixel intensity changes are small, and that intensity *I* is directly proportional to concentration *C* in 2D Cartesian and axisymmetric cylindrical coordinates:

4 $$D\approx \frac{\frac{{{\Delta }}_{t}I}{{\Delta }t}}{\frac{{\Delta }}{{\Delta }r}\cdot \frac{{\Delta }I}{{\Delta }r}+\frac{{\Delta }}{{\Delta }z}\cdot \frac{{\Delta }I}{{\Delta }z}}$$

5 $$D\approx \frac{\frac{{{\Delta }}_{t}I}{\Delta t}}{\frac{{\Delta }}{{\Delta }r}\cdot \frac{{\Delta }I}{{\Delta }r}+\frac{1}{r}\cdot \frac{{\Delta }I}{{\Delta }r}+\frac{{\Delta }}{{\Delta }z}\cdot \frac{{\Delta }I}{{\Delta }z}}$$

where the numerator and denominator terms are approximated discretely (*I*_*t*_, *I*_*r*_ and *I*_*z*_, refer to the intensity value at one space-time coordinate) as follows:

6 $$\frac{{{\Delta }}_{t}I}{{\Delta }t}=\frac{{I}_{t+1}-{I}_{t}}{{\Delta }t}$$

7 $$\frac{{\Delta }}{{\Delta }r}\cdot \frac{{\Delta }I}{{\Delta }r}=\frac{{I}_{r+1}-2{I}_{r}+{I}_{r-1}}{{\Delta }{r}^{2}}$$

8 $$\frac{{\rm{\Delta }}}{{\Delta }z}\cdot \frac{{\Delta }I}{{\Delta }z}=\frac{{I}_{z+1}-2{I}_{z}+{I}_{z-1}}{{\Delta }{z}^{2}}$$

9 $$\frac{{\Delta }I}{{\Delta }r}=\frac{{I}_{r+1}-{I}_{r}}{{\Delta }r}$$

where *Δt* is the time step between stacks, *Δr* is the spacing between each circumference and *Δz* is the spacing between each z plane. For time steps, we compared adjacent time steps only, i.e. 2 & 4 mins, 4 & 6 mins, 6 & 8 mins, etc. (not e.g. 2 & 6 mins, 2 & 8 mins). Then, the average weighted along *r* due to higher variance around the outer space of the area of interest, using the ratio:

10 $$w(r,z)=\frac{1}{{\sigma }^{2}(r,z)}$$

where *w* is the weighted average and *σ*2 is the variance. The weighted average uses the standard deviation (SD) obtained in the previous section $(SD=\sqrt{{\sigma }^{2}})$. Non-real numbers (NaN) are removed and replaced with zeros at this point. This in effect eliminates the centre of the projection holes (saturated zones) from the analysis. As diffusion is a scalar quantity, all remaining numbers were made absolute to avoid potential subtraction of the diffusion coefficient unintentionally when averaging in the next step. The new weighted mean diffusion coefficient is:

11 $${D}_{w}(z)=\frac{{\sum }_{r}(w(r,z)\cdot D(r,z))}{{\sum }_{r}w(r,z)}$$

Repeating this for all time point comparisons of the same patch application/specimen, and assuming diffusivity is independent of time, all comparisons were averaged.

#### Diffusion analysis replicate selection

For replicates containing stacks that did not detect any RD signals, i.e. background noise, those planes were discarded, as they would be falsely indicating a low diffusivity value. This was observed in deeper layers, so approximately 15–20 µm were truncated from the analysis.

## Electronic supplementary material

Figure S1

Figure S2

Figure S3

Figure S4

Supplementary Information

## Data Availability

The datasets generated and/or analysed during the current study are available from the corresponding author on reasonable request.
