# Supplementary material for: Space- and time-resolved investigation on diffusion kinetics of human skin following macromolecule delivery by microneedle arrays
Source: Sci Rep. 2018 Dec 10;8:17759. doi: 10.1038/s41598-018-36009-8 (PMC6288161; doi:10.1038/s41598-018-36009-8)
Supplement: Supplementary file 5 — Supplementary Information [file 41598_2018_36009_MOESM5_ESM.docx]

Jonathan CJ Wei# ^a,b^ and Isha N Haridass# ^c,a^, Michael L Crichton ^d^, Yousuf H Mohammed ^a^, Stefano C Meliga ^b^, Washington Y Sanchez ^a^, Jeffrey E Grice ^a^, Heather AE Benson ^c^, Michael S Roberts* ^a,e^, Mark AF Kendall* ^f^

*Space- and time-resolved investigation on diffusion kinetics of human skin following macromolecule delivery by microneedle arrays*

^a^ Diamantina Institute, Faculty of Medicine, The University of Queensland, Woolloongabba QLD 4102, Australia ^b^ Australian Institute for Bioengineering and Nanotechnology, The University of Queensland, St Lucia QLD 4072, Australia ^c^ School of Pharmacy and Biomedical Sciences, Curtin Health Innovation Research Institute, Curtin University, Bentley WA 6102, Australia ^d^ Institute of Mechanical, Process and Energy Engineering, School of Engineering and Physical Sciences, Heriot-Watt University, Edinburgh, EH14 4AS, United Kingdom ^e^ Basil Hetzel Institute for Translational Health Research, School of Pharmacy and Medical Sciences, University of South Australia, Adelaide SA 5011, Australia ^f^ Australian National University, Canberra ACT 0200, Australia

#These authors contributed equally and share lead authorship.

*Corresponding authors: [m.roberts@uq.edu.au](mailto:m.roberts@uq.edu.au), [mark.kendall@anu.edu.au](mailto:mark.kendall@anu.edu.au),

# Supplementary information


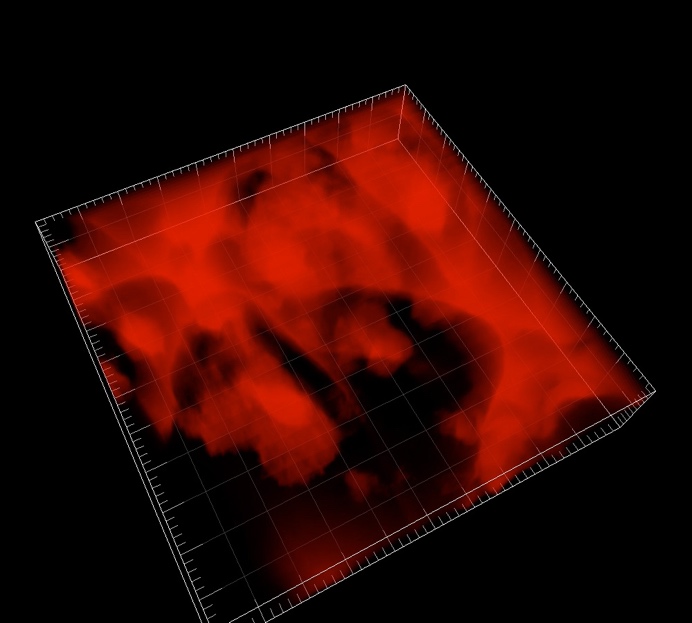

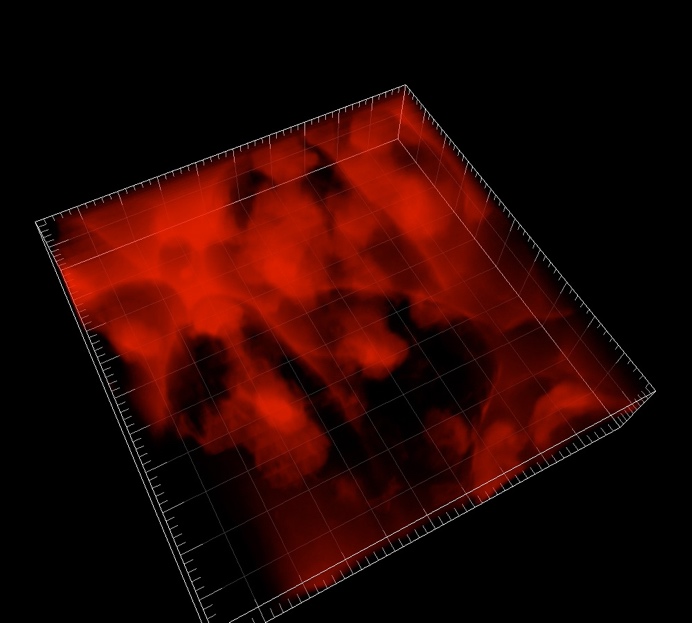


**Figure S 1.** Before and after screenshots of a representative stack visualised in 3D, from 0-30 minutes after patching, red channel, perspective view. Full video attached.


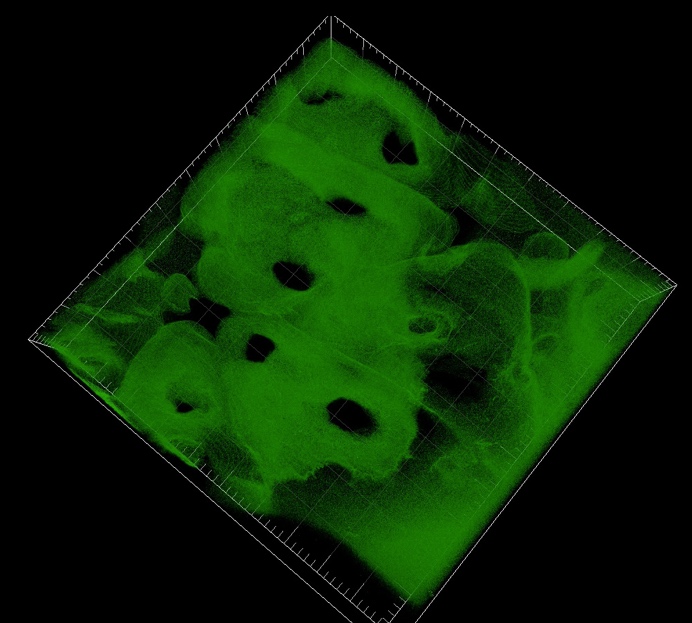

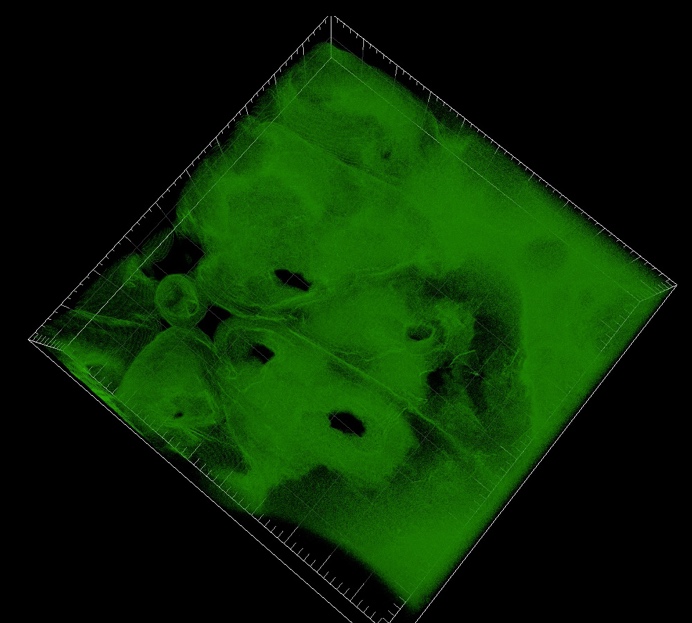


**Figure S 2.** Before and after screenshots of a representative stack visualised in 3D, from 0-30 minutes after patching, green channel, perspective view. Full video attached.


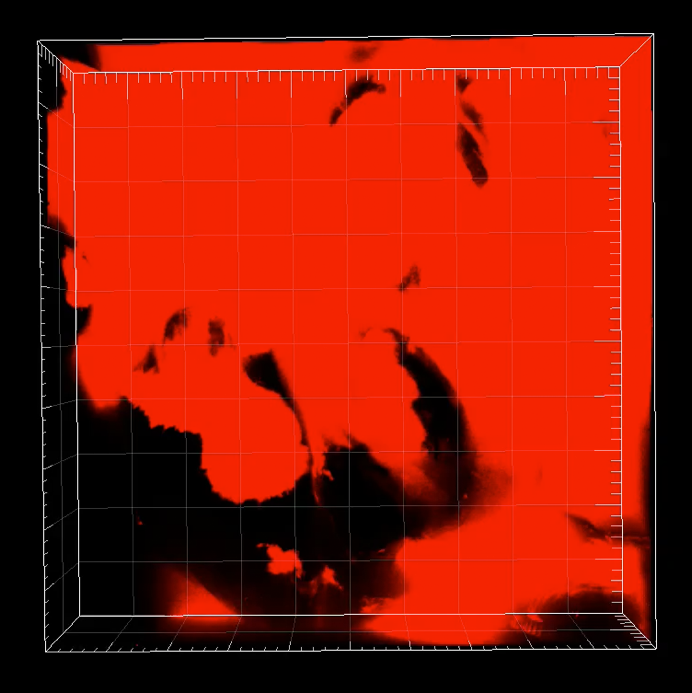

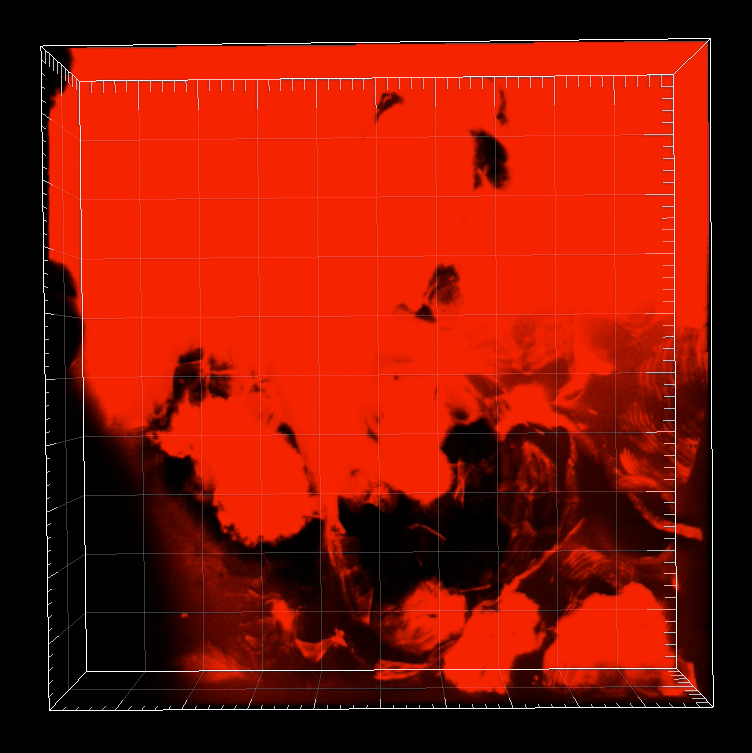


**Figure S 3.** Before and after screenshots of a representative stack visualised in 3D, from 0-30 minutes after patching, red channel, top view. Full video attached.


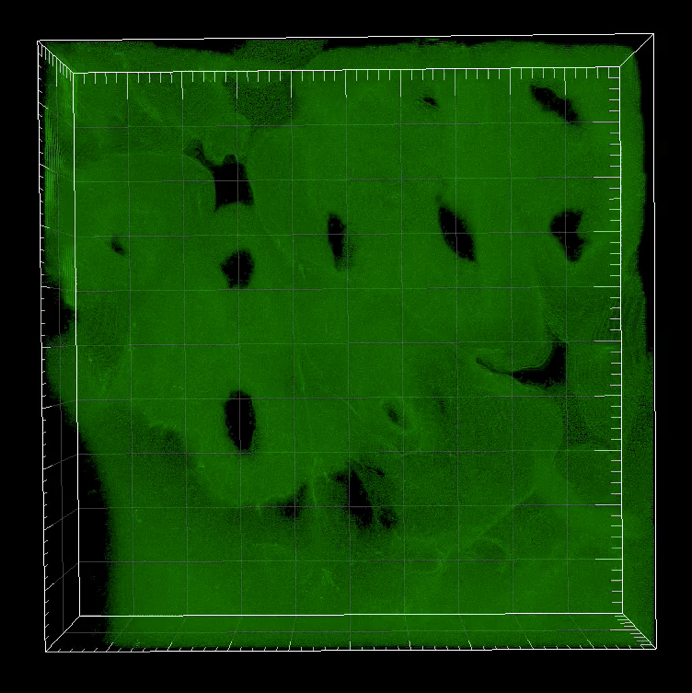

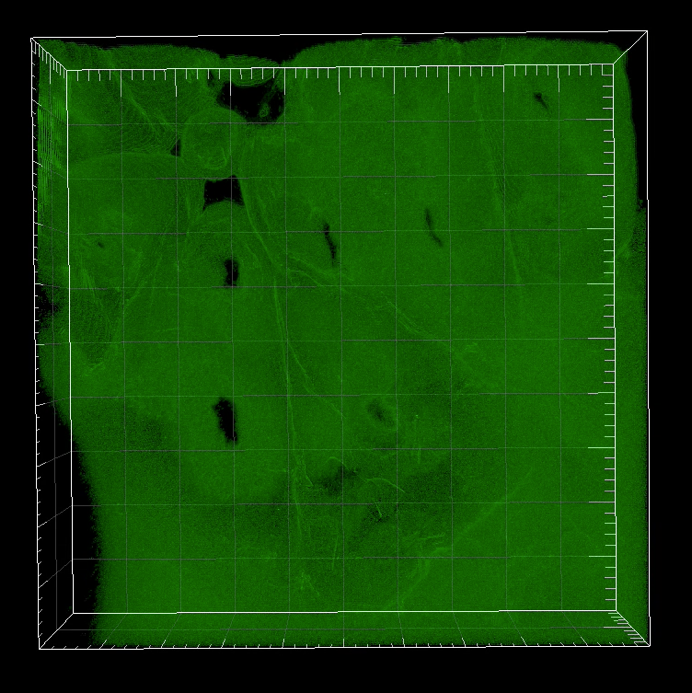


**Figure S 4.** Before and after screenshots of a representative stack visualised in 3D, from 0-30 minutes after patching, green channel, top view. Full video attached.
